# Barriers and Breakthroughs in Targeting the Oxytocin System to Treat Alcohol Use Disorder

**DOI:** 10.3389/fpsyt.2022.842609

**Published:** 2022-02-28

**Authors:** Andrey E. Ryabinin, Yangmiao Zhang

**Affiliations:** Department of Behavioral Neuroscience, Oregon Health & Science University, Portland, OR, United States

**Keywords:** oxytocin, alcohol use disorder, alcoholism, addiction, pharmacotherapy, oxytocin receptor agonist, individualized medicine

## Abstract

Development of better treatments for alcohol use disorder (AUD) is urgently needed. One promising opportunity for this development is the potential of targeting the oxytocin peptide system. Preclinical studies showed that administration of exogenous oxytocin or, more recently, stimulation of neurons expressing endogenous oxytocin lead to a decreased alcohol consumption across several rodent models. Initial clinical studies also showed that administration of oxytocin decreased craving for alcohol and heavy alcohol drinking. However, several more recent clinical studies were not able to replicate these effects. Thus, although targeting the oxytocin system holds promise for the treatment of AUD, more nuanced approaches toward development and application of these treatments are needed. In this mini-review we discuss potential caveats resulting in differential success of attempts to use oxytocin for modulating alcohol use disorder-related behaviors in clinical studies and evaluate three directions in which targeting the oxytocin system could be improved: (1) increasing potency of exogenously administered oxytocin, (2) developing oxytocin receptor agonists, and (3) stimulating components of the endogenous oxytocin system. Both advances and potential pitfalls of these directions are discussed.

## Introduction

Alcohol use disorder (AUD) is a devastating condition where the affected individuals continue to engage in drinking despite the negative experience and the harm caused by drinking. Globally, more than 5% of all deaths have been attributed to alcohol consumption (1). In addition to alcohol-related mortality, AUD is associated with a plethora of debilitating health and societal consequences. Harmful alcohol consumption increased during the COVID-19 pandemic (2–4). Better treatments for AUD with better access to these treatments are urgently needed.

**Figure 1:**
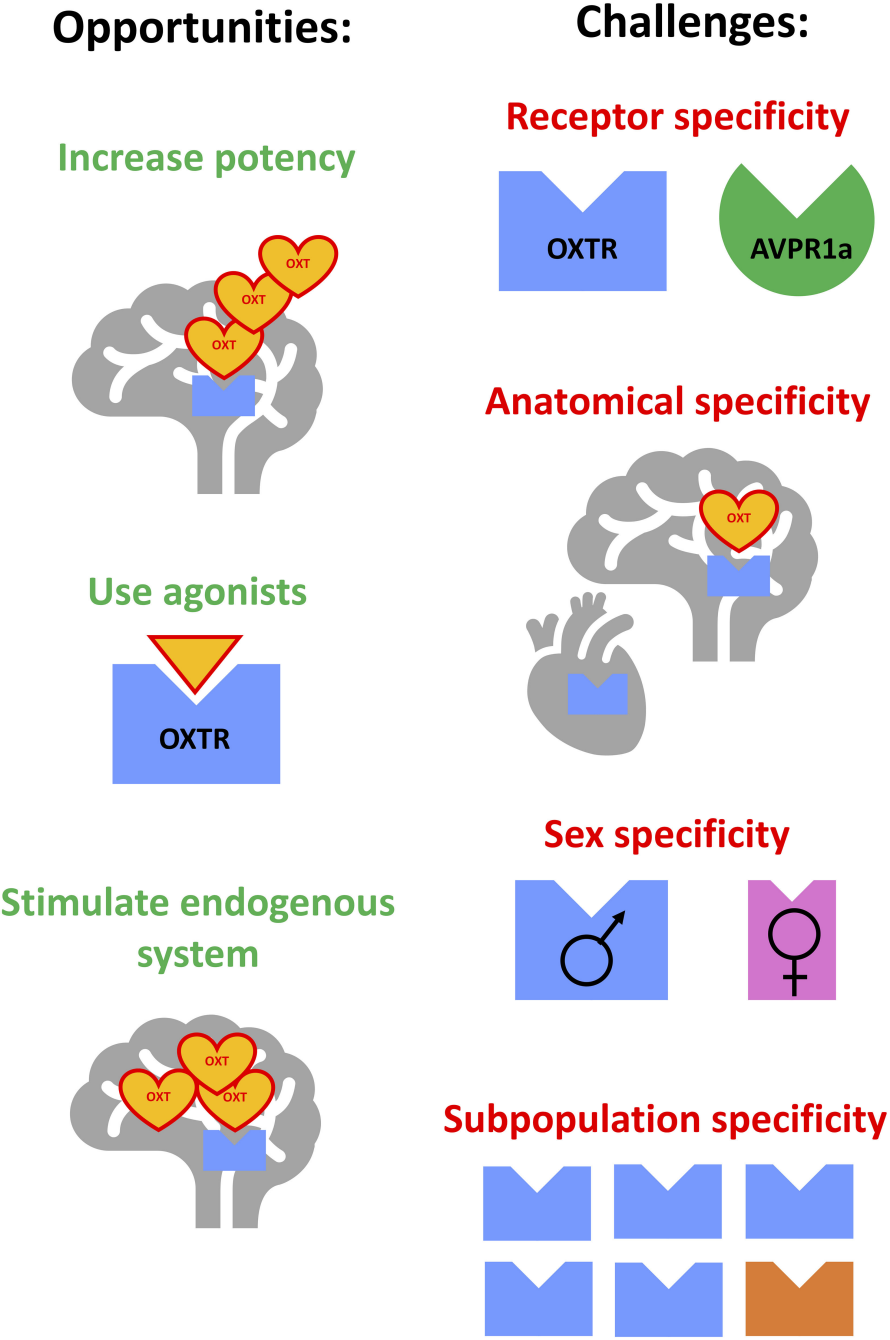
Opportunities and challenges for improving the targeting of the oxytocin system for the treatment of alcohol use disorder. Opportunities: (1) increasing potency of exogenously-administered oxytocin, (2) development and use of oxytocin receptor agonists, (3) stimulating components of the endogenous oxytocin system. Challenges: (1) achieving specificity to oxytocin receptor vs. other related receptors, (2) achieving anatomical specificity (central vs. peripheral, or targeting specific subpopulations of neurons), (3) overcoming or focusing on potential sex-dependent effects, (4) identifying subpopulations sensitive to treatments due to genetic or environment-induced differences.

Among potential targets for such treatments, recent attention has focused on the oxytocin (OXT) neuropeptide system. OXT has a leading role in coordinating maternal behaviors, and regulates other functions, including social attachment, anxiety, food consumption, learning, body temperature, neuroinflammation, and pain (5, 6). OXT has been suggested to modulate alcohol-related responses including craving, tolerance, abstinence, and withdrawal-induced anxiety and social engagement (7–10). In turn, alcohol can affect OXT levels and underlying neurocircuitry (11–13). These observations suggested that stimulating the OXT system could curb excessive alcohol use by targeting several phases of the addiction cycle: modulating alcohol's effects during the intoxication phase, decreasing alcohol's toxicity during the abstinence/withdrawal phase and/or decreasing craving during the anticipation/preoccupation phase (14–17). Indeed, OXT administration decreased alcohol self-administration in numerous preclinical studies (16, 18–21). Importantly, OXT was effective in translationally relevant animal models, including being administered via intranasal (IN) routes, in models of severe alcohol dependence (22), in prairie voles, which share similarities in neurocircuitry regulating social behaviors (21), and in presence of untreated peers, similarly to medication administration in outpatient clinics (23, 24). Initial clinical studies also indicated that OXT administration in humans could decrease signs of withdrawal and craving and inhibit excessive alcohol drinking (12, 17, 25). However, more recent studies had limited success (26–28), calling for more nuanced approaches for the treatment of AUD with OXT. Moreover, recent basic science studies revised our understanding of organization of the OXT system (29) and exogenous OXT's penetrance into the brain (30, 31). With these developments, the goal of this minireview is to critically analyze the potential pitfalls associated with clinical studies testing effects of OXT on signs of AUD (Table 1) and to evaluate different directions of overcoming these pitfalls: by increasing potency of exogenously administered OXT, by developing OXT receptor (OXTR) agonists, and by stimulating the endogenous OXT system (Figure 1).

**Table 1:** Clinical studies on effects of oxytocin administration on alcohol-related behaviors.

**References**	**Population**	**Dose**	**Effects on alcohol craving and intake**	**Other effects**
Pedersen et al. (17)	USA, heavy drinkers, 11: 9M/2F	2 × 24 IU/day × 3 days	↓Penn Alcohol Craving Scale (g~2.7)	↓ Several alcohol WD ratings, ↓ dose of BZ, ↓ anxiety on Profile of Mood States
Mitchell et al. (32)	USA, heavy drinkers, 32: 19M/13F, CO	1 × 40 IU	↓ or ↑ craving (Alcohol Urge Questionnaire) dependent on social attachment anxiety	↓ Approach to appetitive stimuli
Pedersen et al., (25)	USA, heavy drinkers, 22: 13M/9 F	3 × 40 IU/2 days, then 2 × 40 IU /day for 12 weeks	↓ Number of heavy drinking days (g up to~1.9), ↓ drinks/drinking day (g up to ~5.8), lower efficacy at the end. No effect on Penn Alcohol Craving Scale	
Hansson et al. (12)	Germany, heavy drinkers, 12 M, CO	1 × 24 IU	↓ fMRI response to alcohol cue across several brain regions	
Vena et al. (28)	USA, social drinkers, 35: 19M/16F, CO	1x (40 IU + 20 IU)	No effect on subjective and physiological responses to alcohol, including “want more” on Drug Effects Questionnaire	
Bach et al. (33)	Germany, social drinkers, 13 M, CO	1 × 24 IU	↓ Alcohol cue-induced fMRI connectivity (g up to ~1.6), correlated with subjective cue-induced craving ratings	
Flanagan et al. (26)	USA, PTSD/AUD, 67 M	1 × 40 IU	No effect on subjective craving ratings after Tier Social Stress Task	↓ Cortisol response to Tier Social Stress Task
Stauffer et al. (34)	USA, 47—PTSD/AUD, 37—controls, M, CO	1 × (20 IU, 40 IU or placebo)	No effect on subjective cue-induced craving ratings and cue-induced heart rate response	
Melby et al. (35, 36)	Norway, heavy drinkers, 40: 29M/11F	2 × 24 IU/ 3 days	No effect on self-reported alcohol intake and no effect on phosphatidylethanol levels	Not effect on WR questionnaires and BZ for WD, no effect on actigraphy and sleep
Bach et al. (37)	Germany, social drinkers, 18 M, CO	1 × 24 IU	↓ fMRI response to faces (g up to ~4.1), correlated with alcohol craving and heavy drinking days	
Melby et al. (27)	Norway, heavy drinkers, 40: 29 M/11F	2 × 24 IU/ 3 days, then SA up to 24 IU/day for 25 days	No effect on Alcohol Craving Questionnaire and several measures of drinking	↓ Self-reported nervousness
Melkonian et al. (38)	USA, PTSD/AUD, 73 M	1 × 40 IU		↓ Anger in subjects with low subjective rating of craving

*M, male; F, female; CO, cross-over; x, times; IU, international unit; SA, self-administration; WD, withdrawal; BZ, benzodiazepine; red font, decrease; Orange font, decrease in a correlated measure; green font, increase; g, Hedges effect size, estimated where possible*.

## Clinical Studies on OXT Administration and Motivation to Consume Alcohol

So far, 11 clinical studies have been published on effects of IN OXT on alcohol drinking-related behaviors, and one study on social stress response but not drinking-related behaviors in individuals with AUD (38) (Table 1). These studies assessed effects of OXT treatments ranging from single to 12 week-long daily administrations across populations in three different countries, in subjects characterized as social drinkers, heavy drinkers or patients with post-traumatic stress disorder/AUD comorbidity, with modest sample sizes ranging from 11 to 84. Only half of the studies evaluated effects of OXT in both sexes, and most studies focused on males only. Evaluating Table 1, one can conclude that effectiveness of OXT to decrease relevant outcomes was not overwhelming, revealing the following potential pitfalls.

### Insufficient Treatment Period

The majority of studies that looked at short-term outcomes failed to observe decrease in drinking. The only study that reported decreased drinking administered OXT for the longest time (25). Importantly, decreased measures of intake in this study only started to emerge on the second week of treatment. Therefore, the usual duration of OXT treatment and observation in most current studies might be insufficient to show its effect. On the other hand, effects of OXT on measures of heavy drinking in this study appeared to be subsiding in the last weeks of treatment, suggesting either a desensitization to repeated treatment or a “floor effect” of OXT.

### Insufficient and Inconsistent Dosage

It appears that more recent studies, showing less consistent effects, used slightly lower doses of OXT per day. One study relied on subjects' self-administration of OXT, which might have not achieved effective uptake (27). It is important to note that in some of the studies, OXT did not decrease measures of alcohol craving or drinking despite having significant effects on measures of stress or anxiety, arguing against insufficient OXT uptake (26, 27). On the other hand, it could be that higher doses are needed to decrease alcohol drinking than doses that can affect stress or anxiety.

### Individual Variance in the Population

Another consideration is that OXT could be effective only in a subset of patients. One study indicated that OXT can even increase craving in subjects with low attachment anxiety (32). Thus, subpopulations of subjects can be differentially sensitive to OXT treatment, requiring bigger sample sizes to achieve the power necessary to detect effects of these treatments.

In studies listed in Table 1, five studies observed decreases in craving, alcohol cue response or heavy alcohol use (12, 17, 25, 33, 37), and the other five did not observe significant effects (26–28, 34–36). Since only one study reported that OXT produced opposite from hypothesized effects in a subpopulation of subjects (32), we conservatively interpret this analysis that OXT can be effective in decreasing alcohol craving or intake, but its potency needs to be enhanced.

## Enhancing Exogenous OXT's Ability to Decrease Alcohol Drinking

While observations of positive effects of OXT on alcohol drinking in clinical studies are not consistent, this contrasts with a large body of preclinical studies showing effects. Decreased alcohol intake following OXT administration has been observed in mice, rats, and prairie voles. These studies have been previously reviewed (8, 9, 14, 15, 25, 39–41). The success of preclinical studies may offer clues and opportunities to potentiate the effect of OXT treatment in humans.

Non-human studies have explored both peripheral (e.g., subcutaneous, intraperitoneal, and IN) and central routes of administration of OXT to understand its mechanisms of action. Since OXT's half-life in plasma is estimated to be on the order of minutes (42, 43), the observation that peripherally administered OXT had longer effects than expected from its half-life was attributed to the potential feed-forward release of OXT from magnocellular neurons (44, 45).

To overcome the difficulty interpreting results from peripherally administered OXT, some investigators resorted to study its effects following intracranial administration (12, 46, 47). While these studies demonstrated OXT's central modulation of alcohol-related behaviors, they did not clarify how the peptide reaches the relevant intracranial sites. More recent studies indicated that exogenously administered OXT indeed reaches selected brain regions in mice, rats, monkeys and humans (30, 48–52). Since OXT has additional affinity to vasopressin (AVP) receptors, it is worth noting that the exogenously administered and centrally active OXT could also exert its functions via actions that do not involve OXTRs (29, 53, 54).

The IN route results in penetration of a portion of total administered OXT to the brain via several pathways: transmission along the olfactory and trigeminal neural bundles, absorption from basal epithelia directly into cerebrospinal fluid, crossing the blood-brain barrier, and through circumventricular organs (6, 52). The different relative size of the neural bundles vs. whole brain across species might contribute to differences in the access to specific brain regions in rodents vs. humans. Nevertheless, attempts to increase penetration of IN OXT are underway, including the use of mucoadhesives, nanoemulsions and liposomal formulations (6, 55). Another development is the recent recognition that brain penetrance of OXT is aided by the participation of the Receptor for Advanced Glycation End-products (RAGE) (31, 56). Development of allosteric modulators of RAGE could be an interesting approach to enhance the potency of exogenous OXT.

Finally, as mentioned, OXT could be differentially effective across different populations of subjects. OXT was found to differentially affect alcohol craving in subjects with low and high attachment anxiety (32). Several indicators suggest that it could be more effective in males vs. females (57). Moreover, subjects carrying polymorphisms in OXTR and OXT show differences in alcohol consumption patterns (58, 59). Carriers of these polymorphisms could be differentially sensitive to OXT treatments. Studies targeting these subpopulations could be a step toward development of individualized AUD treatments.

## Development of More Potent and Efficacious OXTR Agonists

OXT's lack of stability, low brain penetrance and non-specific effects on AVP receptors prompted researchers to consider developing OXTR agonists (14, 60). The majority of developed and tested OXTR agonist are peptides, with presumably minimal penetrance of the blood-brain barrier. Because of this, peptide-based OXTR agonist PF-06655075 was used to test whether effects from manipulating the OXT system are through central or peripheral OXTRs (22). This is an important consideration as stimulation of peripheral OXTR can affect certain behaviors, for example fear conditioning (61). However, central, but not peripheral administration of PF-06655075 decreased alcohol drinking in dependent rats, indicating that stimulation of the central OXT system is crucial for moderating alcohol consumption (22).

Carbetocin is a highly stable OXT analog used in clinics to prevent postpartum hemorrhage. It has been demonstrated to modulate central effects of morphine (62, 63). In relation to alcohol, however, reported effects are mixed. While the same dose of repeated carbetocin inhibited acquisition, enhanced extinction and suppressed reinstatement of ethanol-induced conditioned place preference in a mouse study (64), it enhanced ethanol-induced conditioned place preference in another study (65). We are not aware of any reports on carbetocin's effects on alcohol drinking. Other peptide OXTR agonists include Lipo-oxytocin-1 and [Thr_4_,Gly_7_]OXT (66– 68). Their effect on alcohol-related behaviors have not been reported, but the strong possibility of effects on various peripheral systems could be a matter of concern.

Another possibility is to explore the effects of OXT metabolites—OXT fragments composed of shorter chained peptides. As smaller molecules, they should have better penetrance into the brain and, therefore, higher potency. In contrast to carbetocin, OXT (4–9) improved social preference in the BALBc/J model of autism spectrum disorder-like social deficits (69). In early studies, the tripeptide OXT (6–9) was shown to attenuate tolerance to hypothermic effects of repeated ethanol injections in mice (70). However, whether OXT metabolites can affect alcohol drinking or alcohol reward is still to be discovered.

Development of non-peptide OXTR agonists continues to progress. Three small-molecule agonists have been reported (14, 60, 71, 72). TC-OT-39 has an EC_50_ of ~100 nM at OXTR as an agonist, but also a moderate affinity at V1a AVP receptors as an antagonist. Therefore, its neurochemical mode of action *in vivo* is difficult to estimate. In any case, it was not effective in studies of BALBc/J model of autism spectrum disorder-like social deficits (69). Comparing to TC-OT-39, WAY-267464 is a level of magnitude more potent OXTR agonist, but with a similar affinity to V1a AVP receptors (71), again making it difficult to evaluate specificity of its effects after systemic administration. Promisingly, however, administration of this compound into amygdala facilitated extinction of fear conditioning (73) whereas systemic injection of this compound rescued not only social deficits in Shank3b mutant mice (74), but also social deficits caused by adolescent alcohol exposure in rats (75).

LIT-001, the most recently developed small molecule, has an agonist activity on OXTR similar to that of WAY-267464 in *in vitro* studies, but several magnitudes lower affinity to AVP receptors, for the first time allowing receptor-specific systemic pharmacology of OXTR (71). Systemic administration of LIT-001 rescued deficits in social interaction in the Oprm1 knockout model of autism spectrum disorder (71) and inhibited inflammation-induced hyperalgesia in rats (76). Studies testing LIT-001 in models of AUD are underway.

Development of small-molecule OXTR agonists is a promising avenue for future research. Though striving to develop compounds with more specific and potent effects than OXT, one has to keep in mind the potential side effects. For example, a compound that has potent anxiolytic and analgesic properties must be evaluated for its own potential addictive properties.

## Harnessing the Endogenous OXT System

OXT is an evolutionary old peptide. Therefore, its mixed affinities toward OXTR and AVP receptors, as well as its poor brain-penetrance, could have advantages for fitness. It might be, therefore, more prudent to stimulate the endogenous OXT system to produce beneficial effects instead of manipulating the system exogenously. As a proof of this concept, a recent study by King et al. used a chemogenetic approach to activate OXT neurons of the mouse paraventricular nucleus of hypothalamus (PVN) (77). This activation significantly decreased alcohol consumption in a mouse model of binge drinking. This finding implicates the need to explore the feasibility to develop effective means of stimulating the endogenous central OXT system for AUD treatment.

The importance of OXT in regulating social attachments is well-demonstrated across vertebrate species (78–83). Positive social interactions are known to stimulate OXT neurons (84–87). Therefore, social interactions could stimulate the OXT system and hence, decrease alcohol consumption. However, the relationship between social interaction and activity of OXT system is more complex. OXT neurons are also activated during social isolation and after exposure to other stressors (88–90). Stressful experiences are well-known to increase alcohol consumption (7, 91, 92). Therefore, it would be more relevant to selectively stimulate OXT system in a way that mimics the effect of positive social interactions to regulate AUD-related phenotypes.

The latter task is extremely difficult because there are currently no methods allowing to evaluate when specific components of the central OXT system are activated in humans. Our understanding of the relationship between the activation of subpopulations of OXT neurons and OXT's peripheral levels continues to change, and knowledge of projections sites of parvocellular and magnocellular OXT neurons are being revised (29). Additionally, it is difficult to estimate the relationship between levels of OXT in plasma vs. levels in the brain. A meta-analysis across 17 studies found no significant associations between peripheral and central—in either cerebrospinal fluid or specific brain regions—OXT levels under basal conditions in both humans and non-human animals (93). On the other hand, this meta-analysis confirmed significant positive associations between these levels after IN OXT in humans and rodents (49, 94, 95) and after experimental stress in rodents (93, 96–98), indicating that correspondence in OXT levels can be established. Recent studies also complicate the interpretation of previous studies showing that alcohol consumption decreases OXT levels (9). For instance, while one study showed significantly higher OXT levels in blood of patients with AUD (99), another study found significantly lower OXT levels in AUD patients (100). The reason for such opposite results needs to be further studied.

Taken together, stimulation of endogenous OXT system could contribute to treatments of AUD by modulating social relationships or adherence to therapy. This potential could be especially relevant to group-based approaches to treatment. However, direct behavioral manipulation of this system to moderate excessive alcohol intake in AUD patients at this point is still a theoretical goal. Its realization relies on better understanding of the OXT system.

## Conclusions

While recent clinical studies show mixed results in the effectiveness of IN OXT to decrease excessive drinking, more sophisticated approaches targeting the OXT system are needed. One possibility is to enhance access of exogenously administered OXT to relevant brain regions either through development of better delivery systems or through enhancement of transporters. Another possibility is to further explore the development of OXTR agonists. The potential of behavioral stimulation of parts of the endogenous OXT system should also be investigated. The better we understand the organization of the OXT system, the greater chance we have of harnessing it for the treatment of AUD.

## Author Contributions

AR and YZ conceptualized and wrote the manuscript. All authors contributed to the article and approved the submitted version.

## Funding

This work was supported by NIH R01 grants (AA019793, AA025024, and AA028680) to AR.

## Conflict of Interest

The authors declare that the research was conducted in the absence of any commercial or financial relationships that could be construed as a potential conflict of interest.

## Publisher's Note

All claims expressed in this article are solely those of the authors and do not necessarily represent those of their affiliated organizations, or those of the publisher, the editors and the reviewers. Any product that may be evaluated in this article, or claim that may be made by its manufacturer, is not guaranteed or endorsed by the publisher.
